# Overweight, Obesity, and Neuropsychological Performance: Results From the Women’s Interagency HIV Study

**DOI:** 10.1093/geroni/igab046.667

**Published:** 2021-12-17

**Authors:** Elizabeth Vasquez, Mark Kuniholm, Leah Rubin, Anjali Sharma, Kathleen Weber, Margaret A Fischl, Michael Plankey, Deborah Gustafson

**Affiliations:** 1 University of Albany, University of Albany, New York, United States; 2 University at Albany (SUNY), Rensselaer, New York, United States; 3 Johns Hopkins University School of Medicine, Baltimore, Maryland, United States; 4 Albert Einstein College of Medicine, Bronx, New York, United States; 5 Cook County Health, Chicago, Illinois, United States; 6 Sylvester comprehensive cancer center, Miami, Florida, United States; 7 Georgetown University, Washington, District of Columbia, United States; 8 SUNY Downstate Health Sciences University, Brooklyn, New York, United States

## Abstract

Conflicting associations of body mass index (BMI) and waist circumference (WC) with neuropsychological performance (NP) are observed in the general population and among people living with HIV. We examined BMI and WC in middle-aged women living with HIV (WLWH) and without HIV (HIV-) in relation to 10-year trajectories of NP in the Women’s Interagency HIV Study (WIHS). NP assessments occurred biennially from 2009-2019. Demographically-adjusted T-scores were calculated for six NP domains: learning, memory, executive function, processing speed, attention and working memory, and motor function. Multivariable linear models stratified by HIV serostatus examined whether baseline (2009) BMI and WC were associated with NP domains - 1) cross-sectionally and 2) longitudinally over 10 years. The sample included 432 WLWH and 367 HIV- women, >40 years old. Most women (73%) were overweight (BMI=25-29.9kg/m2) or obese (BMI=>30kg/m2). Among WLWH, 28% were overweight, 45% obese; among HIV- women, 26% were overweight; 56% obese. Cross-sectionally at baseline, WLWH who were overweight versus normal weight (BMI=18.5-24.9kg/m2), performed worse on executive function, processing speed, and motor function (all p<0.05). HIV- women who were overweight versus normal weight performed worse on memory, learning, executive function, processing speed and motor function (all p<0.05). Baseline BMI and WC were not associated with worsening NP domains in this younger, primarily overweight and obese sample of WLWH or HIV- women (all p>0.05).Future follow-up of these women will enhance understanding of the age when total and/or central obesity may influence NP trajectories and health of the aging brain.

